# The evolving landscape of enzymatic technologies for precision synthesis of antibody–drug conjugates

**DOI:** 10.1093/abt/tbag020

**Published:** 2026-04-29

**Authors:** Thuc Oanh Hoang, Arshad J Ansari, Yong Zhang

**Affiliations:** Department of Pharmacology and Pharmaceutical Sciences, Alfred E. Mann School of Pharmacy and Pharmaceutical Sciences, University of Southern California, Los Angeles, CA 90089, United States; Department of Pharmacology and Pharmaceutical Sciences, Alfred E. Mann School of Pharmacy and Pharmaceutical Sciences, University of Southern California, Los Angeles, CA 90089, United States; Department of Pharmacology and Pharmaceutical Sciences, Alfred E. Mann School of Pharmacy and Pharmaceutical Sciences, University of Southern California, Los Angeles, CA 90089, United States; Department of Chemistry, Dornsife College of Letters, Arts and Sciences, University of Southern California, Los Angeles, CA 90089, United States; Norris Comprehensive Cancer Center, University of Southern California, Los Angeles, CA 90089, United States; Research Center for Liver Diseases, University of Southern California, Los Angeles, CA 90089, United States

**Keywords:** antibody–drug conjugate, ADP-ribosyl cyclase, CD38, enzyme, targeted therapy

## Abstract

Precise control over how and where small-molecule drugs are covalently attached to monoclonal antibodies is increasingly vital for creating consistently efficacious and safe antibody–drug conjugates (ADCs) as powerful targeted therapies. Enzymatic conjugation methods have gained considerable attention for their abilities to efficiently install payloads at defined locations under mild and predictable conditions. This work provides a comprehensive review of current enzymatic technologies for site-specific ADC construction, organized by the biological origin of enzymes. Among various enzyme-based conjugating approaches, a special focus is given to the emerging ADP-ribosyl cyclase–enabled ADC (ARC-ADC) platform. By utilizing genetically fused CD38, a member of the ARC family, together with its dinucleotide-derived inhibitor, site-specific ADCs with defined drug-to-antibody ratios in varied formats could be facilely produced with demonstrated efficacy and specificity in preclinical models of different types of cancer. Unlike most enzymatic methods requiring recognition tags or external catalytic steps, ARC-ADC provides a fully integrated, modular strategy for streamlined ADC discovery and development.

## Teaser

Statement of Significance This work reviews currently established enzymatic approaches for generating homogeneous antibody–drug conjugates (ADCs) with defined drug-to-antibody ratios and highlights a recently developed ADP-ribosyl cyclase-based strategy as a modular platform for integrated synthesis of site-specific ADCs in distinct formats for diverse therapeutic applications.

## Introduction

Antibody–drug conjugates (ADCs) constitute a class of targeted therapeutics that harness the specificity of monoclonal antibody and high potency of cytotoxic drugs to selectively eliminate cancer cells with limited systemic side effects. The conceptual foundation of targeted cellular toxicity was first proposed by Paul Ehrlich in 1913 as the “magic bullet” theory [1] and an ADC delivering methotrexate by leukemia-targeting antibodies was the first experimental prototype for this concept [2]. Decades of advances in antibody discovery, protein engineering, linker chemistry, and payload design [3] ultimately led to the first ADC approval, gemtuzumab ozogamicin (Mylotarg) in 2000 for CD33-positive acute myelogenous leukemia (AML), followed by 14 additionally approved ADCs and hundreds more candidates registered for clinical trials across diverse oncology indications [4, 5].

The aim to broaden therapeutic windows of small-molecule drugs by ADCs, including both reducing minimal effective dose and increasing maximal tolerated dose (MTD), was strongly supported by animal data (e.g. ADC carrying mertansine verse free mertansine [6] and ADC with monomethyl auristatin E (MMAE) verse free MMAE [7] in rodent and nonhuman primate models). However, clinical evidence remains less definitive [6, 7]. A meta-analysis of nearly 40 ADCs across 7 classes of payloads indicated improved efficacy but no markedly enhanced levels of tolerance at normalized MTD in comparison to their free payload counterparts [8]. Systemic toxicities from released drugs and lack of significant improvements in patient outcomes compared with standard chemotherapies are also among the main reasons for the discontinuation of many ADC candidates in clinical trials (e.g. Rova-T and TAK-264) [9–11] or even withdrawals post market approvals (e.g. Mylotarg and Blenrep [12–14]). As a result of nonspecific drug conjugation, ADC heterogeneity is one of key factors that impede successful clinical translation from preclinical research. The wide variability in regioisomers and drug-to-antibody ratio (DAR) in ADC can lead to varied pharmacokinetics, reduced therapeutic index, and unpredictable toxicity profiles [3, 15–17]. Therefore, ADCs with defined DARs and drug attachment sites are increasingly considered to provide superior therapeutic outcomes [3, 17–20].

Site-specific conjugation strategies include chemical methods [21], affinity peptide-directed Fc modifications [22, 23], and enzymatic productions [24, 25], each offering distinct advantages and limitations. Using biorthogonal reactions and engineered amino acid residues (e.g. site-selective mutation/modification and disulfide rebridging [20]), chemical strategies offer versatility and scalability but may require extensive optimization to balance chemical reactivity with antibody integrity. The introduction of mutation, non-canonical amino acids, and reconstructed disulfide bonds also pose risks of altered stability, affinity, specificity, immunogenicity, and biocompatibility for antibody scaffolds. Affinity peptide-directed Fc modification exploits short peptides with Fc-binding selectivity to guide chemical conjugation at defined sites [23, 26]. Similarly, affinity ligands can direct payloads to form noncovalent interactions with antibodies to produce homogeneous ADCs [27]. This affinity-based approach improves selectivity and reduces conjugation-site heterogeneity without requiring genetic engineering of antibodies, while maintaining relatively straightforward reaction conditions. Nevertheless, the use of affinity peptides/ligands faces requirements of binding domains, restrictions on numbers and locations of conjugated payloads, and potential reduction of binding affinity upon payload attachments. By contrast, enzymatic approaches leverage intrinsic selectivity of protein catalysts to achieve precise modifications under mild conditions with demonstrated genetic encodability, catalytic turnover, orthogonality, modularity, and biocompatibility.

This work reviews currently established technologies for enzyme-aided synthesis of ADCs, with a focus on the ADP-ribosyl cyclase-enabled ADC (ARC-ADC) platform. The diversity of available biocatalysts, their amenability to protein engineering as well as advantages and challenges associated with each approach are presented. Various types of enzyme-derived systems along with their conjugation mechanisms, developmental stages, and clinical applications were first summarized. The design, generation, and preclinical evaluation of ARC-ADCs by utilizing the CD38 enzyme, a member of the ARC family, were then discussed, revealing this self-contained antibody–CD38 fusion as a unique and versatile strategy for developing site-specific ADCs with translational potential.

## Enzyme-based platforms for ADC synthesis

**Figure 1:**
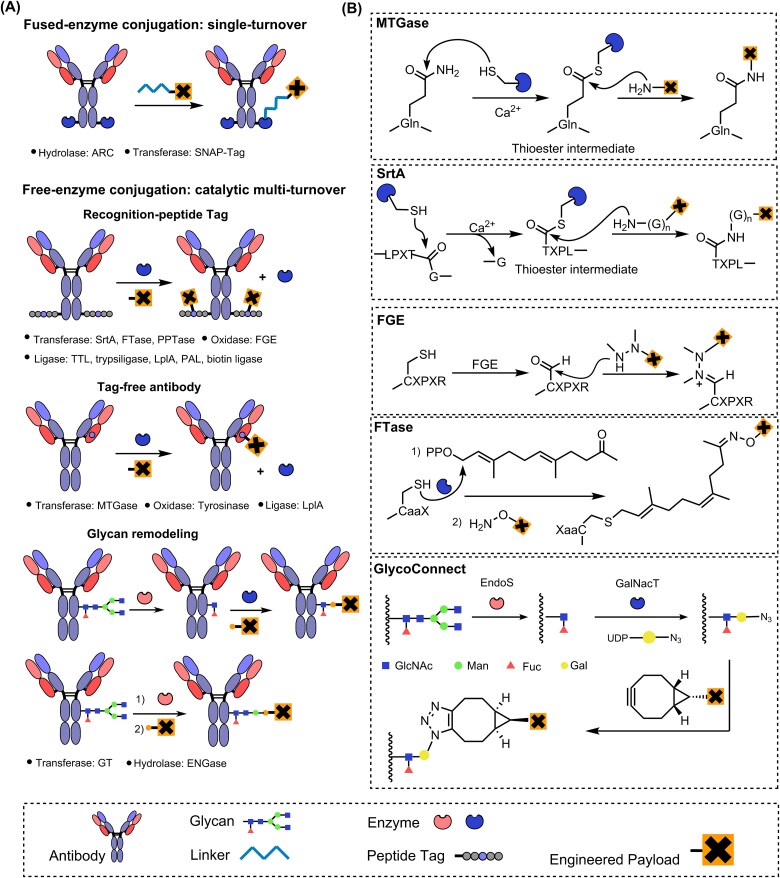
Schematic illustration of enzyme-enabled technologies for precision ADC synthesis. (A) Two major enzymatic conjugation strategies and list of enzymes in each group. (B) Chemistry of enzymatic conjugation approaches at clinical stages.

Enzyme-mediated conjugation has emerged as a powerful strategy for generating site-specific ADCs with defined DARs and enhanced profiles of pharmacokinetics, safety, and efficacy in comparison to conventional chemical methods. Mechanistically, these conjugating tools fall into two main modes of action: self-labeling tags that form covalent bonds with drug molecules and catalytic machineries that recognize peptide motifs to install payloads (Fig. 1). The development platforms can also be categorized according to enzyme origins from bacteria, archaea, fungi, plants, and mammals, reflecting a broad range of catalytic reactions leveraged to achieve controlled, selective, and biocompatible conjugations (Table 1). The following sections highlight representative enzymes from each category and their contributions to the advancement toward next-generation ADCs.

**Table 1:** Enzyme-based approaches for ADC development

Enzyme name	Conjugation mechanism	Disease; pipeline; format; payload	Advantage and limitation
Bacterial enzyme			
Microbial transglutaminase (MTG)	Catalyzes transamidation between primary amine-containing payload and Gln295 in Fc [28, 29] or fused glutamine donor sequence [30] Multi-turnover	HER2^+^ breast and gastric cancer [28, 30] Preclinical and clinical phase 3 [35, 37] IgG, scFv, Fab, and VHH [28–30] MMAE and MMAF [28, 30, 31]	Easy enzyme production and widely used in industry Requires deglycosylation for tag-free antibodies May modify additional Gln residues causing heterogeneity
Endoglycosidase (ENGase)	Removes *N*-glycan from Fc (often leaves a single GlcNAc), enabling transfer of glycan–drug or modified glycan for conjugation [32] Multi-turnover	Breast and ovarian cancer [33, 34] Clinical phase 1 and 3 [40, 82] IgG [33] Pyrrolobenzodiazepine, MMAE, mertansine, and exatecan [32, 35]	High specificity with minimal impact on antibody sequences May need multistep workflow
Formylglycine-generating enzyme (FGE)	Converts cysteine in fused CXPXR to formylglycine for conjugation [36, 37] Multi-turnover	Breast cancer and skin squamous cell carcinoma [38] Preclinical and clinical phase 1 [82] IgG [36] and scFv [38] MMAE [36, 38]	A small tag with flexible sites Relatively slow reaction rates
Sortase A (SrtA)	Performs transpeptidation between C-terminally fused LPETG and oligoglycine-modified payload [39] Multi-turnover	Breast [40] and ovarian [41] cancer, melanoma [42], and non-Hodgkin lymphoma [40, 43] Preclinical and clinical phase 1/2 [58, 59] IgG [39, 41–43] and Fab [44] MMAE and anthracycline [41, 44, 45]	A small tag Forms native peptide bonds Reversible reactions causing low yields
Phosphopantetheinyl transferase (PPTase)	Transfers phosphopantetheinyl group from CoA-payload to fused serine tag [24] Multi-turnover	HER2^+^ breast cancer [46, 47] Preclinical IgG [46, 47] MMAF [46, 47]	A small tag with flexible sites Reduced efficiency for bulky CoA derivatives
Lipoic acid ligase A (LplA)	Generates amide linkage between lysine in fused LAP-tag and lipoic acid-payload or adds lipoic acid derivative to Lys188/190 in Fab for payload conjugation [48] Multi-turnover	HER2^+^ breast cancer [49] Preclinical IgG [48] MMAE [48–50]	Requires optimal ATP concentrations for conjugation Reversible reactions
Archaeal, fungal, and plant enzyme			
Biotin ligase	Attaches biotin derivative to specific lysine in fused peptide tag for drug conjugation [24] Multi-turnover	HER2^+^ cancers [51] Preclinical IgG MMAE	A medium-size tag Slow catalytic turnovers Requires two-step conjugation
Tyrosinase	Oxidizes selective tyrosine in antibody to *o*-quinone for rapid reaction with nucleophile-containing payload [52] Multi-turnover	HER2^+^ cancers [53] Preclinical IgG MMAE	Requires no tag and mild conditions Off-target oxidation Reactive intermediates may lead to undesired reactions
Peptide asparaginyl ligase (PAL)	Ligates peptides at defined Asn/Asp motif for payload conjugation [54–56] Multi-turnover	Pancreatic, colorectal, gastric, and non-small cell lung cancer Preclinical IgG [54]	A small recognition motif Fast kinetics
Mammalian/human enzyme			
Farnesyltransferase (FTase)	Prenylates C-terminal fused CaaX motif for payload conjugation [24, 57] Multi-turnover	Breast cancer [57, 58] Clinical phase 1 [81, 145] and 3 [82] IgG MMAF [58, 59]	A small recognition motif Requires C-terminal fusion
Glycosyltransferase (GT)	Transfers modified sugars to glycan in Fc for drug conjugation [60] Multi-turnover	HER2^+^ cancer and lymphoma [61] Clinical phase 1 and 3 [87] IgG Doxorubicin, MMAE, and exatecan [32, 61, 62]	Recognizes various sugars High specificity Multistep glycan engineering
Tubulin tyrosine ligase (TTL)	Adds tyrosine derivative-payload to C-terminal fused Tub-tag [63, 64] Multi-turnover	Non-Hodgkin lymphoma [64, 65] Preclinical IgG [64, 65] and nanobody [63] MMAE [64]	A small Tub-tag Requires C-terminal fusion and ATP
ADP-ribosyl cyclase (ARC)	Catalyzes attachment of NAD^+^ derivative-payload to fused CD38 [66] Single-turnover	Breast [66] and prostate cancer [67] and leukemia [68] Preclinical IgG [66–68] and Fab [69] MMAF [66–68]	High specificity and fast reaction rates Flexible and multiple fusion sites Relatively large fusion
Highly engineered enzyme			
O6-alkylguanine-DNA alkyltransferase (AGT; SNAP-tag)	Transfers O6-benzylguanine derivative to Cys145 of fused SNAP-tag for payload conjugation [70] Single-turnover	Triple negative breast cancer [71] and EGFR^+^ cancer [72] Preclinical scFv [71, 72] MMAF [71]	Fast reactions Relatively large fusion
SpyLigase	Produces isopeptide linkage between SpyTag and KTag, fused or conjugated with antibody or payload [73, 74] Multi-turnover	Breast cancer [73] Preclinical IgG MMAE	Forms stable isopeptide bonds Requires two tags Slow turnovers
Intein	Ligates and self-cleaves whole intein or split intein fused to antibody/payload [24] Single-turnover	Breast cancer Preclinical IgG [75] MMAF	Traceless tags Requires reduced conditions and relatively complex fusions
Trypsiligase	Cleaves fused YRH-tag and ligates to payload bearing RH-moiety [76] Multi-turnover	Preclinical Fab [77, 78] Mertansine [71, 77]	A small recognition tag Reversible reactions

### Bacterial enzymes

*Microbial transglutaminase* (MTGase) is a Ca^2+^-independent acyltransferase that forms an isopeptide bond between a glutamine residue and a primary amine. As a small single-domain enzyme, MTGase is easier for production and *in vitro* applications than mammalian transglutaminases. The most common MTGase used in generating ADCs originates from *Streptomyces mobaraensis* [79]. The active-site cysteine in MTGase can attack Gln295 on deglycosylated IgG, forming a thioester intermediate to react with a nucleophilic amine from the payload–linker conjugate. The resulting homogeneous ADCs feature a DAR of 2. Through genetic engineering, this system enables production of ADCs from fully glycosylated antibodies [28, 80], antigen-binding fragments (Fab), single-chain variable fragment (scFv), and variable heavy domain of heavy chain (VHH) [30]. However, MTGase can crosslink endogenous glutamine and lysine residues, producing high-molecular-weight species that complicate ADC manufacturing. By completely removing the C-terminal lysine, a major crosslinking site, the levels of high-molecular-weight variants in final products could be substantially reduced [81]. This platform can also be adopted into two-step chemo-enzymatic processes, in which MTGase first catalytically adds a spacer to the antibody and then the linker-payload is installed via click chemistry [29]. MTGase is a Generally Recognized as Safe (GRAS) material by the FDA and widely used in food industry for working in a wide range of temperature and pH levels. The phase 3 ADC candidate DP303 utilizes MTGase-mediated coupling of an amine-bearing MMAE to the Gln295 site of a monoclonal antibody targeting human epidermal growth factor receptor 2 (HER2) and exhibits high stability, favorable pharmacokinetics, and strong efficacy [31, 82–86].

*Endoglycosidases* (ENGases) such as Endo-S and Endo-S2 from *Streptococcus pyogenes* are hydrolases that cleave Fc *N*-glycans between the two *N*-acetylglucosamine (GlcNAc) residues of chitobiose cores on host IgG antibodies as a mechanism to evade the immune clearance [32]. To facilitate drug conjugation, ENGases were engineered to efficiently remove native *N*-glycans and transfer synthetic glycans with functionalized tags onto the exposed GlcNAc residue of antibodies without hydrolyzing the resulting transglycosylation products [87]. This catalytic transfer step enables precise incorporation of azido, alkyne, and other bioorthogonal handles for downstream drug attachments. Zhang *et al*. established a glycan-derived conjugation platform using Endo-S2 and clickable disaccharide oxazolines to generate homogeneous ADCs with defined DARs from 2 to 12, showing potent cytotoxicity against HER2-positive cell lines [88]. In addition, ENGases can be combined with other types of enzymes for ADC development. Sadiki *et al*. employed Endo-S2 to remove the Fc *N*-glycans, facilitating MTGase-catalyzed conjugation at Gln295 of cetuximab [33]. Endoglycosidases are coupled with glycosyltransferases in Synaffix’s GlycoConnect technology to produce IBI343, an anti-Claudin antibody armed with exatecan (DAR = 4). It revealed promising activities in multiple phase 1 clinical trials and is advancing into phase 3 for gastric cancer [32, 89].

*Formylglycine-generating enzymes* (FGEs) recognize a short peptide motif containing cysteine (CXPXR) and oxidize it into a formylglycine [90], a conserved post-translational modification across all domains of life. Bacterial FGEs such as those from *Mycobacterium tuberculosis* and *Streptomyces coelicolor* are well studied and have become valuable tools in antibody conjugation [91]. With an antibody fused with the CXPXR sequence, FGE generates a formylglycine residue featuring a unique aldehyde functional group for site-specific chemical attachments of payloads. Because of this aldehyde functionality, the engineered peptide sequence is commonly described as an “aldehyde tag.” Several bioconjugation chemistries including hydrazino-iso-Pictet–Spengler (HIPS) coupling and Knoevenagel ligations have been optimized for attaching hydrazide- and pyrazolone-containing cytotoxic agents onto these aldehyde sites, yielding defined drug-to-aldehyde tag ratios of 1:1 and 2:1, respectively [25]. Using this approach, ADCs against epidermal growth factor receptor (EGFR) [36, 38, 92, 93] and CD22 [94] have been generated. TRPH-222, a CD22-targeted antibody with maytansine conjugated through the FGE strategy, showed encouraging outcomes in a phase 1 clinical study for B-cell lymphoma [94].

*Sortase A* (SrtA) is a cysteine transpeptidase belonging to the cysteine protease family, predominantly found in the membrane of Gram-positive bacteria such as *Staphylococcus aureus* [95]. It catalyzes a transpeptidation reaction between an antibody engineered with a pentapeptide recognition motif LPXTG (most commonly LPETG) and a drug linker carrying an N-terminal oligoglycine motif, producing homogeneous ADCs. During SrtA catalysis, a thioacyl-enzyme intermediate is formed between its Cys184 residue and the LPXTG motif, followed by nucleophilic attack from the amino group of (Gly)_n_-payload substrate to complete drug conjugation and SrtA release [41]. Using the SrtA-based approach, anti-CD20 ADCs with MMAE were generated for targeting B-lineage lymphomas [25, 43, 96]. In addition to synthesizing potent ADCs via one-step conjugation by SrtA, a two-step conjugation method exploiting bifunctional oligoglycine linkers was established to produce ADCs with high DARs. Due to relatively low catalytic efficiency, excess amounts of (Gly)_n_–drug conjugate and SrtA enzyme are required to ensure adequate levels of conjugation. Additionally, SrtA is combined with the affinity-driven conjugation technology and other enzymes to increase DARs [97] or attach different types of payload to the same antibody [54]. Manufactured through SrtA-mediated conjugation, an ADC NBE-002 targeting receptor tyrosine kinase-like orphan receptor 1 (ROR1) has reached to the clinical trial [45, 98].

*Phosphopantetheinyl transferases* (PPTases) are essential enzymes for activation of acyl- and peptidyl-carrier proteins (ACPs and PCPs) in bacterial fatty acid and non-ribosomal peptide synthesis by transferring a 4′-phosphopantetheine group from coenzyme A (CoA) to a conserved serine residue in ACP and PCP domains [25]. To utilize PPTases for bioconjugation, Walsh *et al*. developed short 11- and 12-residue peptide tags that can be recognized by PPTases [99, 100], avoiding the need of fusing large ACP and PCP domains with 80–100 residues. Among PPTases, Sfp from *Bacillus subtilis* and AcpS from *Escherichia coli* are the most widely studied for bioengineering applications. Sfp was shown with superior efficiency in recognizing short peptide tags and conjugating a variety of CoA derivatives. Grünewald *et al*. inserted the same 11- and 12-mer tags into trastuzumab at various loop sites for auristatin conjugation catalyzed by Sfp. The produced 95 homogeneous ADCs almost all showed subnanomolar potency against HER2-positive cell lines and those carrying payloads at constant region 1 of the heavy chain displayed favorable pharmacokinetics [46, 47].

*Lipoic acid ligase A* (LplA) from *E. coli* is an ATP-dependent ligase that catalyzes the adenylation of lipoic acid and subsequent transfer to a lysine residue on its native protein substrates [50]. In the context of bioconjugation, a 13-residue lipoic acid-accepting peptide (LAP: GFEIDKVWYDLDA) has been developed for genetic fusion into an antibody and recognition by LplA to generate an amide linkage between the lysine sidechain and carboxyl group of lipoic acid [48]. LplA variants (e.g. W37V) were created to improve ligation activity and promiscuity for lipoic acid derivatives [48, 101]. After installation of modified lipoates onto LAP tags by LplA, cytotoxic payloads could be attached via bioorthogonal reactions. Interestingly, recent studies identified Lys188 in IgG1 for preferential modifications by LplA without the need of LAP tags, resulting in anti-HER2 and anti-CD20 ADCs [49, 102]. Additionally, LplA was combined with MTGase to conjugate two different fluorescent payloads on separate sites of trastuzumab for demonstration of orthogonal cargo releases [103].

### Archaeal, fungal, and plant enzymes

*Biotin ligases* found in various species including bacteria (*E. coli*), archaea (*Pyrococcus horikoshii*), and fungi (*Saccharomyces cerevisiae*) catalyze a two-step ATP-dependent reaction in which biotin is transferred to a specific lysine residue of a biotin acceptor domain [51]. Clickable biotin analogues have been developed for catalysis by yeast and archaeal biotin ligases [104]. To generate site-specific ADCs, a p67 tag derived from the C-terminal sequence of human propionyl-CoA carboxylase α subunit was genetically fused to the C terminus of anti-HER2 trastuzumab light or heavy chain. After site-specifically modifying this antibody-fused tag with desthiobiotin-azide by *P. horikoshii* biotin ligase, MMAE cytotoxic payloads were covalently attached through strain-promoted azide–alkyne cycloaddition [51]. Despite high specificity and compatibility with bioorthogonal chemistry, this modular platform is limited due to ATP dependency, slower catalytic turnovers compared with other ligases, and requirement of expression of the relatively large p67 recognition sequence.

*Tyrosinases* derived from fungi like *Agaricus bisporus* and bacteria such as *Bacillus megaterium* have been used as enzymatic tools for site-specific bioconjugation by oxidizing phenols to highly reactive *ortho*-quinones under mild aerobic conditions [52]. They can transform surface tyrosine residues on the Fc domain (e.g. Tyr296) into *o*-quinone groups for subsequent conjugation with functionalized payloads [105] or convert phenol-drug derivatives to reactive *ortho*-quinone intermediates for coupling with anilines, prolines, and thiols present on antibodies [53]. Cao *et al*. employed the tyrosinase to conjugate phenol-labeled MMAE with anti-HER2 antibodies bearing engineered cysteines for the synthesis of ADCs with DARs of 2–4 [53]. While the tyrosinase-assisted conjugation allows site-specific reactions under mild conditions and needs no genetic incorporation of non-canonical amino acids, only surface-accessible or engineered tyrosine residues are suitable for this approach. In addition to potential off-target oxidation or side reactions, the generated quinone intermediates may lead to crosslinking or undesired reactivities, requiring further investigation and optimization for ADC development.

*Peptide asparaginyl ligases* (PALs) are plant members of the asparaginyl endopeptidase family. Butelase-1 from *Clitoria ternatea* and VyPAL2 from *Viola yedoensis* have been widely studied for protein engineering [55]*.* PAL-catalyzed reactions involve recognition of a tripeptide motif (NHV for butelase-1 and NGL for VyPAL2), cleavage of the peptide bond after the Asn residue, formation of a thioacyl-enzyme intermediate, and ligation with N terminus of another peptide to produce a new peptide bond in high efficiency and minimal hydrolysis [55, 56]. Their key advantages in bioconjugation include rapid reaction kinetics [106], short recognition sequences, and almost traceless modifications with only a single Asn residue left on target proteins post-ligation. PALs have been applied to engineer proteins and peptides with dual functionalities [54, 56]. For example, an EGFR-targeting affibody could be covalently labeled with a fluorescent tag and a cytotoxic peptide through tandem ligation by butelase-1 and VyPAL2 [106]. This method also enabled cyclization of the affibody and subsequent doxorubicin conjugation. ADCs derived from Singzyme’s PAL technology are under preclinical development for different cancers.

### Mammalian/human enzymes

*Farnesyltransferases* (FTases) are heterodimeric prenyltransferases found across eukaryotes. Currently, the best characterized FTases originate from mammals and yeast. FTase catalyzes the transfer of a 15-carbon farnesyl isoprenoid from its prenyl donor farnesyl pyrophosphate onto protein substrates containing a Cys-aliphatic-aliphatic-X motif (CaaX), forming a stable thioether linkage with the cysteine residue of CaaX [107]. Antibodies or antibody fragments engineered with accessible CaaX motifs could be installed with clickable handles by FTases in the presence of azido- or alkyne-functionalized farnesyl analogues for conjugation with cytotoxic payloads via click chemistry [108, 109]. FS-1502, an anti-HER2 ADC carrying monomethyl auristatin F (MMAF) developed by the FTase platform, revealed promising antitumor activity and low toxicity in HER2-positve cancer patients in a phase 1a/1b trial and has entered phase 3 clinical studies [59, 110]. The FTase-based ADC approach features highly specific conjugation via a stable thioether bond under biocompatible conditions with minimal impact on antibody structure.

*Mammalian glycosyltransferases* (GTs), particularly β-1,4-galacto-syltransferases (GalTs) and sialyltransferases, permit incorporation of reactive sugars such as azido- or keto-modified monosaccharides into conserved Fc *N*-glycans at Asn297, providing bioorthogonal handles without altering the antibody backbone [60]. Several preclinical studies demonstrated efficient additions of azido or alkyne groups via GalT-mediated glycan remodeling for attaching cytotoxic payloads and fluorophores [61, 88, 111]. This glycoengineering strategy produced site-specific conjugates of anti-CD22-doxorubicin and anti-HER2-MMAE that can selectively kill cancer cells [61, 112]. By combining an engineered endoglycosidase (Endo-S2) and a native glycosyltransferase, the GlycoConnect technology facilitates the transfer of 6-azido-*N*-acetylgalactosamine (GalNAc) onto Fc glycans for payload conjugation via metal-free cycloaddition, yielding stable site-specific ADCs with improved safety and pharmacokinetics in comparison to conventional conjugation methods. ADCs developed by the GlycoConnect platform are currently in phase 1 (ADCT-601, XMT-1592, and MRG004a) and phase 3 studies (IBI343) [62, 110]. While this conjugation strategy has little impact on antigen binding via spatially distant, well-conserved and chemically distinct glycan sites, extended incubation at 30–37 °C together with modifications on Fc glycans may affect pharmacokinetic profiles and interactions with Fc receptors, prompting further evaluation during ADC development [24, 110].

*Tubulin tyrosine ligase* (TTL) is a post-translational ATP-dependent enzyme widely conserved in all mammals. It catalyzes the addition of tyrosine to C terminus of α-tubulin, generating tyrosinated tubulin critical for regulation of microtubule homeostasis [113]. By employing the TTL, tyrosine derivatives were successfully attached to anti-GFP nanobodies via a Tub-tag (VDSVEGEGEEEGEE) fused at C terminus to mimic the TTL-recognition sequence of α-tubulin [63]. Building on this enzymatic platform, TTL has been applied to develop CD30-specific ADCs against hematopoietic malignancies [64, 65]. TUB-010, an anti-CD30 ADC with MMAE payloads, exhibited superior tumor control compared with Adcetris when administered at equivalent MMAE doses while improving tolerability in both rodent and nonhuman primate models of CD30-positive lymphoma [64].

### Highly engineered enzymes

*SNAP-tag* is a modified version of human O6-alkylguanine-DNA alkyltransferase (AGT) that catalytically transfers the alkyl group from guanine O6 in DNA to Cys145 at the active site for resolving DNA damages [70]. Phage display–assisted screening identified an AGT variant with reduced size, enhanced activity for O6-benzylguanine analogues, and minimized interactions with endogenous DNA [114]. By fusing the SNAP-tag to antibody fragments, benzylguanine-derivatized cytotoxic agents could be covalently attached in a 1:1 stoichiometry [70, 71]. SNAP-tagged anti-EGFR and -HER2 scFvs conjugated with auristatin F payloads revealed potent anti-breast cancer cell activities at nanomolar levels [115]. SNAP-tag-based conjugation has so far been applied to scFv antibodies rather than full-length IgG. It offers the practical advantage of not requiring removal of enzyme after conjugation. A related self-labeling system HaloTag, which fuses an engineered bacterial haloalkane dehalogenase to proteins of interest, operates in a similarly irreversible manner, but has yet to be used to generate ADCs [116].

*SpyLigase* is an engineered enzyme derived from the CnaB2 domain of *S. pyogenes* and split into SpyTag, KTag, and SpyLigase fragments. The sidechains of an aspartic acid residue from SpyTag and a lysine residue in KTag can form a stable isopeptide bond catalyzed by SpyLigase under mild aqueous conditions [24, 117]. Using an anti-EGFR antibody with fused SpyTag at C terminus and a MMAE-KTag peptide, a homogeneous ADC with ~80% conjugation efficiency and a DAR of ~1.7 was synthesized by *E. coli*–expressed SpyLigase and showed subnanomolar cytotoxicity against EGFR-positive cancer cells [73]. Although SpyTag and KTag are small with no integration of SpyLigase into final conjugates, it was engineered primarily to favor stable assembly over rapid release, resulting in low turnover rates and requirement of multiple molar equivalents of enzymes relative to substrates [73]. Similar technologies inspired by the CnaB2-type domain from bacteria but lacking applications toward ADC development include SpyCatcher, SnoopLigase, and Snoop Catcher [74, 118, 119].

*Inteins* found in bacteria, archaea, and eukaryotes can ligate two protein fragments via a native peptide bond through self-splicing or trans-splicing. Through genetic fusion of the whole intein to an antibody, the expressed protein ligation (EPL) strategy can generate a thioester intermediate for the attachment of a synthetic peptide [24]. Based on this method, immunoconjugates with a yield of 60% could be produced [120]. An anti-HER2 antibody conjugated with auristatin F via the streamlined EPL could effectively inhibit tumor growth in a murine xenograft model of breast cancer [75]. While the intein-based conjugation approach results in minimal tag residues on ADCs, it needs to maintain a reducing environment for reactions, causing increased complexity of recombinant expression and modest yields.

*Trypsiligase* is derived from rat trypsin II with mutations to suppress proteolysis while enhancing ligase/transpeptidase activity. By recognizing a YRH motif and cleaving the peptide bond between the tyrosine and arginine residue, trypsiligase catalyzes the formation of an acyl-enzyme intermediate and subsequent ligation of a second peptide with an acyl donor (e.g. 4-guanidinophenyl ester) to the exposed N or C terminus [25]. A homogeneous ADC with cytotoxic payloads could be synthesized by trypsiligase upon fusion of the YRH sequence at C terminus of an anti-HER2 Fab antibody [77]. Compared with other enzymatic methods, trypsiligase requires a short recognition tag for conjugation reactions [24].

## Generation of site-specific ADCs by ARCs

As a member of the ARC family, CD38 enzyme was recently utilized for generating ADCs through genetic fusions with antibodies coupled with its covalent inhibitor carrying cytotoxic payloads. The following section is focused on the coordinated engineering of antibody scaffolds, CD38 enzymatic domains, and dinucleotide-derived covalent inhibitors as drug linkers. These integrated components led to establishment of the ARC-ADC platform that enables facile synthesis of site-specific ADCs with defined DARs. The various designs of ARC-ADCs targeting distinct tumor-associated antigens exhibited marked efficacy and safety across multiple cancer models, demonstrating versatility and therapeutic potential of this conjugation technology.

### CD38 and drug-linker design

CD38 is a multifunctional protein serving as receptor, co-receptor, secreted soluble ligand, and enzyme that plays important roles in intra- and intercellular signaling and metabolism under both physiological and pathological conditions [121, 122]. Structurally, human CD38 is a type II transmembrane glycoprotein including a short N-terminal cytosolic tail, a transmembrane helix, and a large extracellular catalytic domain. Beyond its receptor functions, CD38 is characterized by distinct enzymatic activities within its extracellular domain [123, 124]. Its primary enzymatic function is hydrolysis of nicotinamide adenine dinucleotide (NAD^+^), generating ADP-ribose (ADPR) and nicotinamide [125–128]. CD38 also possesses ADP-ribosyl cyclase activity that converts a minor fraction of NAD^+^ into cyclic ADP-ribose (cADPR). In addition, it can hydrolyze cADPR to ADPR. At acidic conditions such as in lysosomes, CD38 catalyzes a base-exchange reaction, using nicotinamide adenine dinucleotide phosphate (NADP^+^) and nicotinic acid as substrates to produce nicotinic acid adenine dinucleotide phosphate (NAADP). Through degradation of NAD^+^ and production of calcium-mobilizing second messengers including ADPR, cADPR, and NAADP, CD38 ectoenzyme participates in modulation of NAD^+^ metabolism and calcium signaling pathways [129–133].

During CD38 catalysis with NAD^+^, the side chain of Glu226 residue at the active site attacks the anomeric C1′ of the nicotinamide mononucleotide (NMN) moiety to form a covalent intermediate upon dissociation of the nicotinamide leaving group, followed by hydrolysis to yield ADPR or intramolecular cyclization initiated by adenine N1 as a nucleophile for producing cADPR [134, 135]. According to the CD38 catalytic mechanism, covalent inhibitors 2′-F-arabinose NMN (2′-F-araNMN) and 2′-F-araNAD^+^ were developed, which can potently inactivate CD38 through rapid formation of an arabinosyl-ester bond with the catalytic Glu226 residue [136, 137].

To utilize CD38 covalent inhibitors as drug linkers for ADCs, the fluorine atom of 2′-F-araNAD^+^ was replaced by a chlorine to increase covalent bond stability [66]. As structural examinations of CD38 indicated solvent accessibility and lack of critical molecular interactions for the adenine group of NAD^+^ [138], a clickable azido was installed at adenine N6 to serve as a bioorthogonal handle for payload attachments. These designs and considerations led to a new NAD^+^-based inhibitor of CD38, 2′-Cl-araNAD^+^-N_3_, which can block CD38 catalytic activities via a highly stable covalent adduct at Glu226 as validated by enzymatic kinetic assays, mutational analysis, mass spectrometry, and X-ray crystallography [66]. Incubation of CD38 with 2′-Cl-araNAD^+^-N_3_ for 20 min at room temperature results in >90% conjugation efficiency [66]. Moreover, the inherent stability of the pyrophosphate diester of NAD^+^ in circulation [139] combined with its capability of triggering efficient payload release upon cellular uptakes marks this dinucleotide analogue as a promising linker for ADC discovery.

### ARC-ADCs in different formats

**Figure 2:**
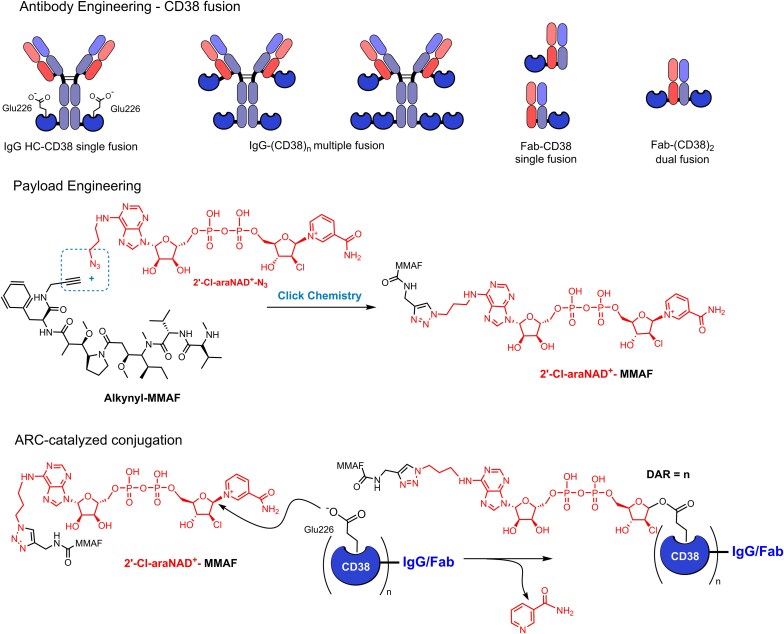
Schematic of generation of ARC-ADCs in varied formats.

The first ARC-ADC was prepared by genetically fusing the CD38 extracellular domain to C terminus of the heavy chain of an anti-HER2 IgG for site-specific conjugation with 2′-Cl-araNAD^+^-MMAF (Fig. 2). In contrast to the N-terminal CD38 fusion, the C-terminal IgG–CD38 fusion showed a strong and comparable CD38 enzymatic activity but a higher binding affinity to the recombinant HER2 antigen. Incubating the 2′-Cl-araNAD^+^-MMAF conjugate with anti-HER2 IgG–CD38 fusion protein overnight on ice produced a site-specific ARC-ADC with a DAR of two. Serum stability and cell-based functional assays indicated adequate stability of the NAD^+^-derived drug linker. Molecular analysis of lysosomal and cell lysates with the anti-HER2-ARC-ADC indicated rapid release of 6-adenine-MMAF. *In vitro* and *in vivo* studies using cellular and animal models of breast cancer demonstrated excellent stability, efficacy, specificity, and safety profiles for this anti-HER2 ARC-ADC [66].

To increase drug loading capacity and efficacy of ARC-ADCs, CD38 catalytic domains were fused to both the heavy and light chains of an IgG antibody specific for C-type lectin-like molecule-1 (CLL-1) [140], a biomarker associated with AML (Fig. 2). After site-specific conjugation with the 2′-Cl-araNAD^+^-MMAF, anti-CLL-1 ARC-ADCs with DARs of two or four were generated [68]. The anti-CLL-1 ARC-ADC carrying four MMAF molecules was shown to display significantly higher potency in killing CLL-positive cancer cells and suppressing AML expansion in mice compared with the anti-CLL-1 ARC-ADC with two MMAF payloads.

Tandem fusion at IgG C termini was then explored to increase numbers of fused CD38 molecules while minimizing impact on the binding of IgG antibodies to tumor antigens. By tandemly fusing CD38 enzymatic domains to heavy and/or light chains of an IgG antibody targeting human prostate-specific membrane antigen (PSMA) for CD38-mediated site-specific conjugation of MMAF tubulin inhibitors [67], anti-PSMA ARC-ADCs with defined DARs of two, four, six, and eight were successfully produced. Interestingly, those internally fused CD38 domains without free N or C terminus still retain robust catalytic activities for conjugating reactions. *In vitro* cytotoxicity studies of anti-PSMA ARC-ADCs indicated the highest potency for the anti-PSMA ARC-ADC with a DAR of six in eliminating PSMA-expressing prostate cancer cells. Despite additionally fused CD38 domains, the anti-PSMA ARC-ADC with a DAR of eight showed lower cytotoxic effects on PSMA-positive tumor cells due to reduced antigen-binding affinity and stability. Animal studies supported significant *in vivo* anti-prostate tumor efficacy for the anti-PSMA ARC-ADC with six MMAF payloads.

In addition to IgG–CD38 fusions, the ARC-ADC technology was extended to Fab antibodies. The CD38 catalytic domain was fused to C terminus of an anti-HER2 Fab heavy and/or light chain, giving rise to Fab fusion proteins with single or dual CD38 molecules (Fig. 2). Consistent with previous findings, C-terminal fusion of CD38 to the Fab antibody causes no negative effects on HER2 antigen binding or CD38 catalytic activity. Using the 2′-Cl-araNAD^+^-MMAF conjugate for incubation with anti-HER2 Fab–CD38 fusions, ARC-enabled Fab–drug conjugates (ARC-FDCs) with DARs of one or two were generated [69]. The anti-HER2 ARC-FDC with dually conjugated MMAF exhibited slightly stronger potency against HER2-expressing breast cancer cells relative to the ARC-FDC with mono MMAF. In addition to improving fractions of the payload, these FDCs with much lower molecular weights than those of IgG-based ADCs may promote tissue penetration and distribution.

### ARC-ADC versus other enzymatic strategies for ADC development

A variety of enzyme-assisted site-specific conjugation strategies have been established to improve ADC homogeneity, control DARs, and streamline manufacturing processes. These enzymatic tools fall into two main categories: (i) multi-turnover free enzymes that attach engineered payloads to antibodies at recognition motifs and (ii) single-turnover fused enzymes that self-react with functionalized payloads to form stable covalent linkages. While multi-turnover enzymes offer flexible engineering options and minimal antibody modifications, their catalytic reactions often require additional cofactors, such as controlled temperature and/or pH, and sometimes suffer from reversible or incomplete conjugations [24, 70]. Furthermore, downstream chromatographic purification is commonly required for removals of free enzymes, which reduces overall yields and complicates production steps. Bacterial enzymes are relatively easy for expression but may require antibody deglycosylation for drug conjugations and carry risks of endotoxin contamination. Despite better biocompatibility, mammalian enzymes are more difficult to produce, and nonhuman recognition tags in ADCs may raise immunogenicity.

In contrast, self-labeling platforms including SNAP-tag and CD38 simplify conjugation and purification because no separation of macromolecular enzymes is required at the completion of conjugations. Both systems allow rapid, high-yield reactions under mild conditions that preserve antibody structures and functions. SNAP-tag, however, is an engineered protein eliciting potential host immune responses and has thus far been limited to the scFv-fusion at a ratio of 1:1 [141–143]. As a native human enzyme with low immunogenicity, CD38 can be tandemly fused with antibodies without compromising catalytic activities, supporting higher DARs and facile and efficient payload conjugations. It is also compatible with different antibody formats for generating site-specific ADCs with tailored pharmacological properties. While fusion of multiple CD38 domains for increased DARs reveals minimal impact on antibody functions, the expression yields of antibody fusions start decreasing with incorporation of additional CD38 molecules. Interactions of the fused CD38 with other receptors and ligands in the body may give rise to off-target effects for ADCs, requiring further safety examination.

## Challenges and perspectives of enzymatic conjugation in biopharmaceutical development

Enzyme-catalyzed conjugation technologies offer significant promise for improving the precision and homogeneity of ADCs, which in turn enhance batch control, simplify product specifications, increase clinical and quality reproducibility, and facilitate regulatory approval. Importantly, several challenges remain for their broader clinical translation. One potential concern is the immunogenicity derived from encoded peptide tags or chemical linkages created by nonhuman enzymes. In addition, alterations to the antibody architecture with recognition tags and/or enzymatic modifications may influence molecular stability, biological activity, biodistribution, or clearance profiles. Consequently, these factors must be carefully evaluated during preclinical development and clinical translation.

Manufacturing and process development require additional considerations for enzymatic conjugation technologies. Compared with conventional conjugation approaches, enzyme-based strategies may introduce new steps for materials and process components, such as biocatalysts, cofactors, engineered recognition tags, and specialized linker-payload substrates. These requirements can increase process complexity and impose extra constraints on reaction optimization and supply chain management. Scalable production further demands precise control of enzymatic reaction parameters, including enzyme concentration, reaction time, and purification procedure, to ensure consistent conjugation efficiency and minimize product-related impurities.

Beyond oncology, enzyme-associated technologies may facilitate the generation of antibody conjugates carrying labeling agents or non-cytotoxic payloads such as immunomodulatory agents, antimicrobial compounds, and enzyme inhibitors [144–146]. It can extend antibody-related conjugates to diagnosis and treatment of infectious, inflammatory, and metabolic diseases. Moreover, the enzymatic strategy can be applied toward site-specific conjugation with non-antibody proteins and bispecific conjugates [124, 147, 148].

## Conclusion

Enzymatic conjugation has been emerging as an elegant approach for producing ADCs with high degrees of precision, consistency, and modularity needed to overcome inherent limitations of conventional chemical methods. By situating the ARC-ADC technology in the broad landscape of enzyme-based ADC platforms, this review highlights current advances and limitations of various catalytic methods for production of site-specific ADCs. The ARC system built upon CD38’s robust and NAD^+^-dependent chemistry provides a uniquely integrated solution to ADC development that eliminates the need for exogenous enzymes, engineered recognition tags, and complex and harsh reaction conditions, thereby simplifying production and improving control over conjugation outcomes.

The ARC-ADC technology holds a great promise for expansion beyond the MMAF cytotoxic payload. The inherent flexibility of NAD^+^-linked chemistry suggests accommodation of a wide array of functional molecules, enabling diverse therapeutic and diagnostic applications. As it advances from laboratory-scale proof-of-concept work to clinical assessments, the scalability and feasibility of chemistry, manufacturing, and controls for CD38-mediated conjugation will be next critical steps. The modular nature of CD38 fusion also opens opportunities for innovative bioengineering designs.

## Data Availability

All data supporting the findings of this work are included in the article.
